# A case report on 2 cases of closed scapular fractures caused by low-voltage alternating current shock

**DOI:** 10.1097/MD.0000000000047431

**Published:** 2026-03-13

**Authors:** Jia-Qi Jia, Hong-Jun Liu, Jun-Chao Dai, Kang Geng

**Affiliations:** a Department of Plastic and Burn Surgery, National Key Clinical Construction Specialty, The Affiliated Hospital of Southwest Medical University, Luzhou, Sichuan, People’s Republic of China; b Department of Anesthesiology, The Affiliated Hospital of Southwest Medical University, Luzhou, Sichuan, People’s Republic of China.

**Keywords:** alternating current, electric injury, low voltage, scapular fracture

## Abstract

**Rationale:**

Scapular fractures caused by domestic low-voltage alternating current (220 V, 50 Hz) electrical injury are rare and can be easily missed or misdiagnosed in emergency clinical practice. This report presents 2 such cases and, through a pooled analysis of relevant literature, summarizes and analyzes the underlying mechanism, key points for early diagnosis, and treatment strategies for this rare injury pattern. The aim is to enhance clinical awareness of this condition and to provide a reference for early recognition and standardized management.

**Patient concerns:**

At the instant of electrical current passage through the upper limb, both patients experienced an intense shock, followed immediately by severe shoulder pain and restricted upper limb movement. Upon presentation, despite only minor cutaneous injuries, both patients reported intense pain localized to the scapular region. This discrepancy between the relatively mild superficial findings and the severity of their symptoms caused considerable confusion and concern, with fear of an invisible severe injury.

**Diagnoses:**

Computed tomography imaging confirmed scapular fractures in both patients.

**Interventions:**

After excluding the risk of potential systemic injuries, both patients underwent conservative management comprising sling immobilization, analgesia, and progressive rehabilitation exercises.

**Outcomes:**

Following 6 weeks of treatment, both patients reported satisfaction with the therapeutic effect, and at the 6-month follow-up, they demonstrated complete functional recovery of the shoulder joint.

**Lessons:**

Even in the absence of visible entry or exit cutaneous lesions, the intense tetanic muscle contractions induced by low-voltage electrical shock can be sufficient to cause scapular fractures. For patients presenting with shoulder pain following low-voltage alternating current electrical injury – regardless of skin integrity – clinicians should maintain a high index of suspicion for this rare injury so as not to miss the diagnosis.

## 1. Introduction

Benefiting from the hidden position and the surrounding muscles, the scapulae are generally not easily damaged. Scapular fractures are quite rare, accounting for approximately 1% of all fractures, and are mostly secondary to high-energy injuries often accompanied by other tissue damage. This also means that simple scapular fractures following household low-frequency alternating current (AC) shocks are much rarer.

Owing to the rare occurrence of such cases, coupled with the deceptive and confusing presentations of electric injuries, some fracture symptoms would not appear at the time of injury, and even the most experienced trauma/burn surgeons find it difficult to ensure that there will be no missed diagnosis.^[1]^ There have been several reports of delayed diagnosis and treatment among the few cases of fractures after electric shock.^[2–4]^ We collected this series of cases and summarized their similarities, trying to highlight the special situation that electric shock may cause scapular fractures and to reiterate the importance of early recognition to arouse the attention of clinicians to such traumas.

## 2. Case presentation

### 2.1. Case 1

A 27-year-old male chef was admitted to the hospital with right shoulder pain and dysfunction following a low-voltage electric shock. The incident occurred when his wet right hand was in close proximity to a live 220 V/50 Hz alternating current terminal. In the absence of direct physical contact, a conductive circuit was established between the moist hand and the power source, allowing current to flow through his right upper limb. After a 4 to 5 second period of exposure, he managed to free his arm. Immediately following the shock, the patient remained conscious and experienced no falls, palpitations, or chest tightness. His sole immediate symptoms were a pronounced sensation of electric shock concurrent with tonic muscular contraction in the right upper limb. Within minutes, the patient reported stiffness in the right limb accompanied by pain in the right scapular region.

**Figure 1.**
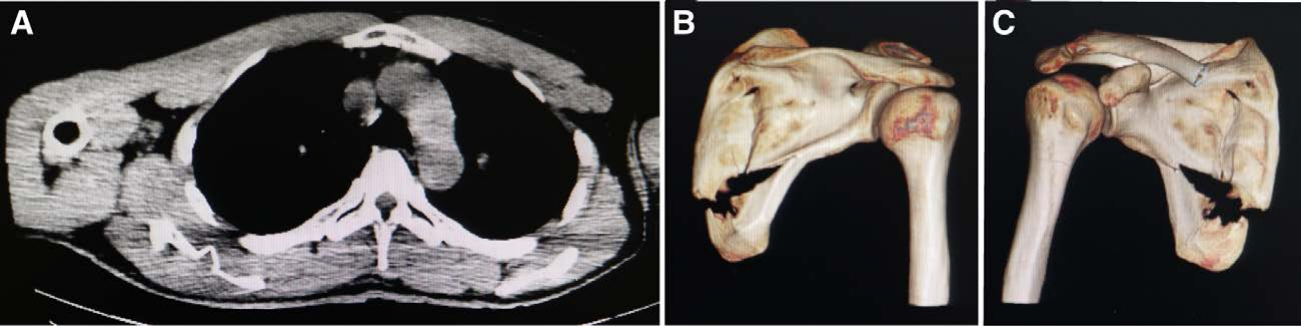
Partial examination results of case 1. (A) Chest CT scans showed a fracture of the right scapula without muscle tear or hematoma. (B, C) CT 3-dimensional reconstruction manifested multiple fractures of the right scapular body with displacement of the broken ends, no joints were damaged. CT = computed tomography.

The results of the laboratory examination were as follows: myoglobin, 163.7 μg/L; creatine kinase-myocardial band, liver and kidney function, and electrocardiogram (ECG) were normal, the computed tomography (CT) scan of the chest suggested a fracture of the right scapula (Fig. 1A). Physical examination results were as follows: blood pressure, 122/82 mm Hg; pulse rate, 88 bpm; respiratory rate, 20 bpm; body temperature, 36.6°C; the mind was clear and the skin was intact; no burns were observed at the entrance and exit; the affected limb felt numb and uncomfortable, and the muscles of the right trapezius, scapula, and shoulder girdle were slightly swollen with obvious tenderness. The patient’s right forearm was limited to lifting and abduction.

After admission, 3-dimensional CT reconstruction of the shoulder joint was performed to evaluate the degree of the fracture. As shown in Figure 1B and C, the patient had multiple fractures of the right scapular body with displacement of the broken ends. We took symptomatic measures, such as oxygen inhalation, ECG monitoring, and analgesia, and immobilized the patient’s shoulder joint with a wide arm sling. The patient was discharged 48 hours later. He recovered well after discharge after 6 weeks of shoulder immobilization, analgesia, and progressive physical therapy. After 6 months of follow-up, the patient returned to normal shoulder joint function.

### 2.1.1. Patient presentation

The patient reported being startled by a sudden flash and a loud “bang” at the moment of the incident. He initially believed his injuries were not serious. Several minutes later, however, he developed persistent pain in his right shoulder, which prompted him to seek medical attention. A subsequent CT scan confirmed a scapular fracture, which the patient found surprising given the brief nature of the electric shock. A telephone follow-up conducted at 6 weeks post-injury documented good recovery of shoulder function. The patient reported satisfaction with the treatment outcome and expressed gratitude to the medical team.

### 2.2. Case 2

A 30-year-old male was admitted to the hospital with left shoulder pain and impaired function following a low-voltage electrical injury. The incident occurred 5 hours prior to admission when the patient accidentally contacted a live 220-Volt household source with his left hand while changing a light bulb at home. He described an immediate sensation of electrical current traversing from his left hand proximally along the upper limb. The electrical contact lasted for approximately 4 to 5 seconds, during which the patient remained conscious and reported no associated traumatic injury, fall, palpitations, or chest discomfort. After briefly sitting down to rest for a few minutes, he experienced a sudden onset of severe, “tearing” pain in his left shoulder, accompanied by a subjective sensation of weakness and stiffness in the affected limb.

**Figure 2.**
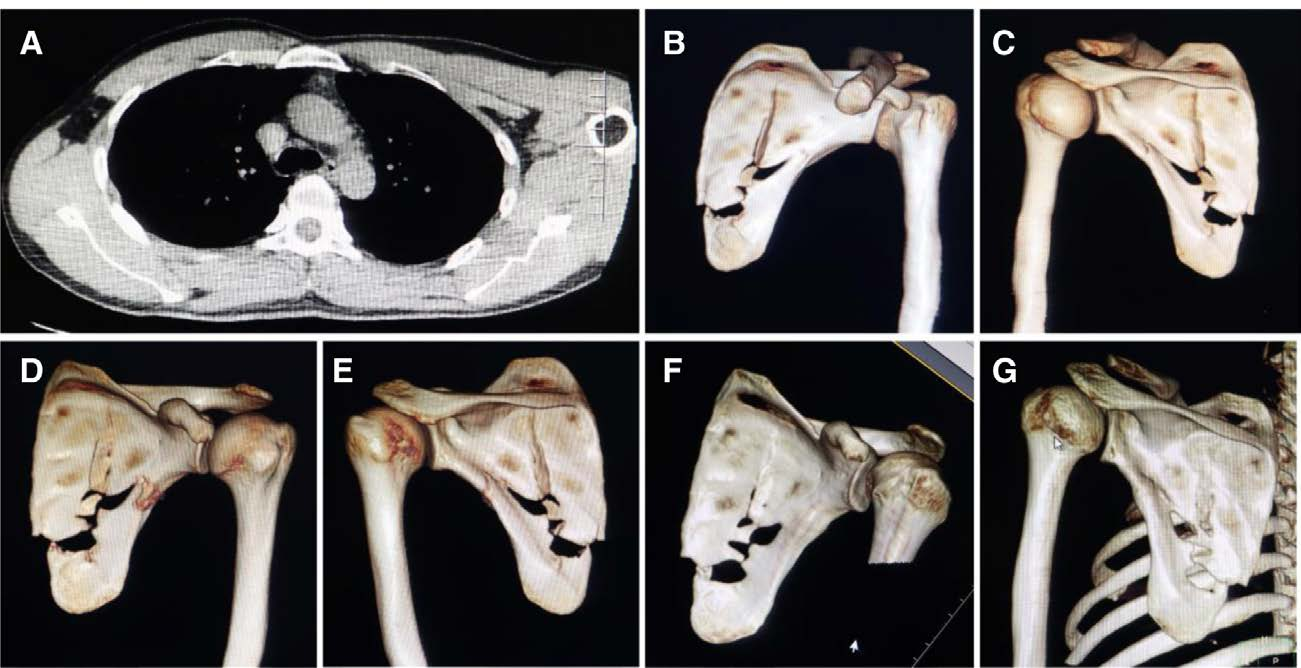
Partial examination results of case 2. (A) Chest CT scans suggested a fracture of the left scapula without muscle tear or hematoma. (B, C) CT 3-dimensional reconstruction pointed to multiple fractures of the left scapular body with displacement of the broken ends, the joints were not involved. (D, E) Calluses were more than before on reexamination 18 days from injury. (F, G) 3D reconstruction images at review 3 months after the accident. CT = computed tomography.

The results of laboratory examination were as follows: myoglobin, 148 μg/L; creatine kinase-myocardial band, 5.3 μg/L; liver and kidney function, ECG was normal; the CT scan of the chest suggested a fracture of the left scapula (Fig. 2A). The physical examination results were as follows: blood pressure, 126/79 mm Hg; pulse rate, 82 bpm; respiratory rate, 21 bpm; and body temperature, 37°C. At the entrance of the current, a slight full-thickness skin burn was noted on the proximal knuckle of the left index finger, presenting as a 5 mm × 5 mm grayish yellow scorched skin. However, the sensation and movement of the index finger were normal. At the exit of the current, there was a slight skin lesion at the distal end of the left-third toe. The patient experienced numbness and discomfort in the affected limb, local swelling and obvious tenderness in the left shoulder, and left upper limb lifting and abduction limitation.

As shown in the 3-dimensional CT reconstruction images, the patient had multiple fractures of the left scapular body with displacement of the broken ends (Fig. 2B and C). Conservative therapy was then initiated. A 48 hours after admission, the patient was discharged. CT 3-dimensional reconstruction of the left shoulder joint at reexamination 18 days later indicated increased calluses of the left scapula (Fig. 2D and E). After 6 weeks of shoulder immobilization with wide arm sling, analgesia, and progressive physical therapy after discharge, the patient recovered well. Three months later, the tenderness in the left shoulder disappeared, and the mobility of the shoulder joint improved significantly (Fig. 2F and G). The patient’s normal shoulder function was mostly restored after 6 months of follow-up.

### 2.2.1. Patient presentation

The patient described a momentary current sensation traversing his body during the electrical contact, which triggered bodily tremors and left him considerably alarmed. Subsequently, he developed severe pain in his left shoulder accompanied by a subjective sensation of weakness and stiffness; these symptoms persisted and ultimately prompted him to seek hospital evaluation. He expressed considerable surprise when informed that CT imaging revealed a scapular fracture. Fortunately, following a period of conservative management, his shoulder function recovered satisfactorily, enabling him to resume normal daily activities and return to work. The patient reported satisfaction with the treatment outcome.

## 3. Discussion

**Figure 3.**
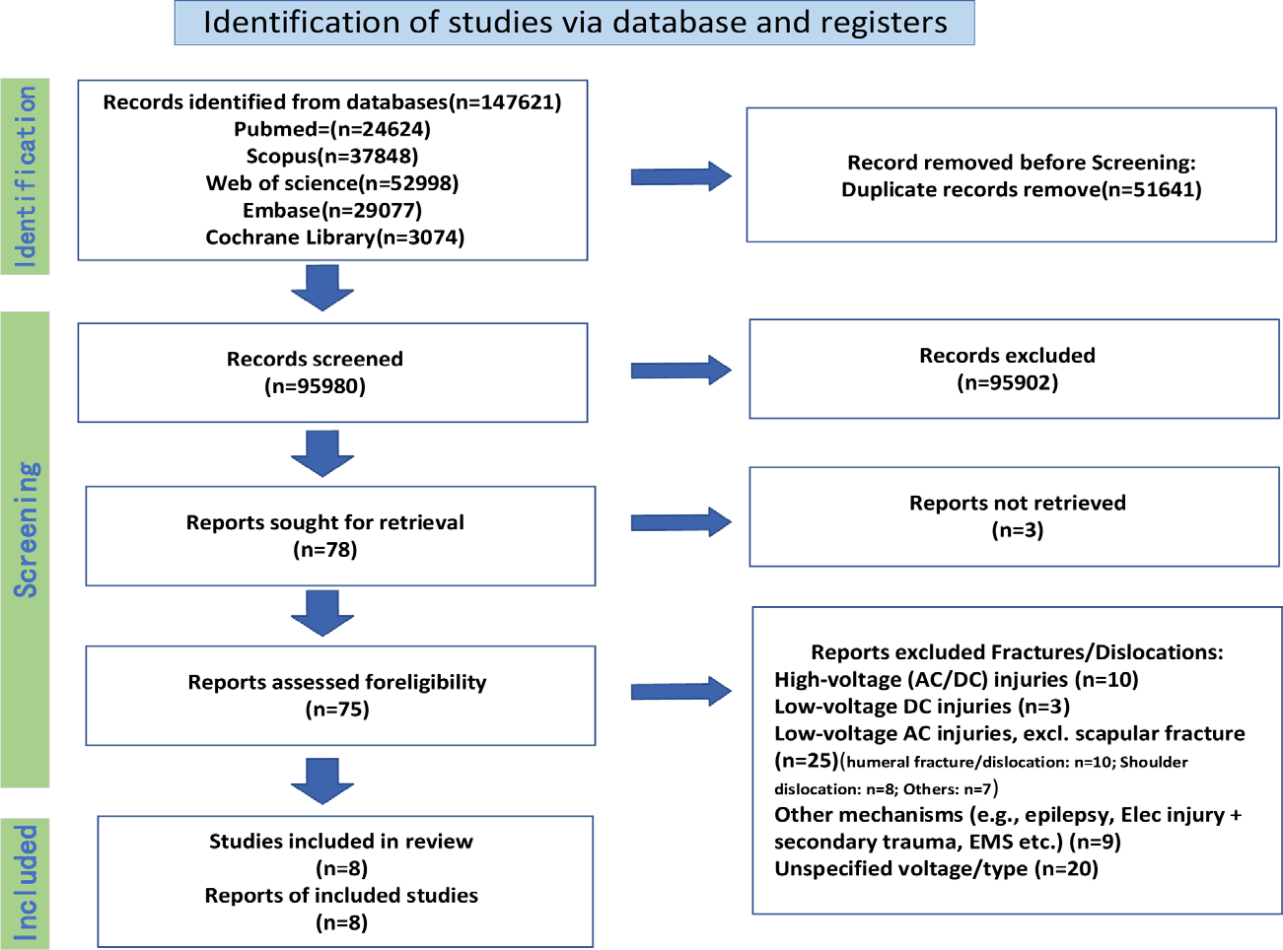
Flowchart of the literature selection process. Inclusion criteria: (1) well-documented history of low-voltage (<1000 V) alternating current electrical injury. (2) Confirmed diagnosis of scapular fracture. Exclusion criteria: (1) combined high-energy secondary trauma. (2) Injuries caused by electrical sources other than low-voltage alternating current (e.g., high-voltage electricity, direct current, lightning and others). (3) Preexisting local scapular pathology or tumor. (4) Unretrievable full text or insufficient data after contacting the original authors. EMS = emergency medical services.

This study reports 2 cases of closed scapular fractures without shoulder dislocation resulting from low-voltage alternating current electrical injuries. Both cases presented 2 critical and shared characteristics: 1st, the fractures occurred under low-energy trauma conditions; and 2nd, the fractures were isolated to the scapula itself. This distinctive injury pattern prompted us to investigate the relationship between low-voltage alternating current shock and specific types of fractures. To contextualize our findings within the broader literature, we conducted a systematic review. We searched 5 databases (PubMed, Scopus, Web of Science, Embase, and Cochrane Library) for relevant studies from their inception until October 2020, using the keywords “electric shock,” “electrocution,” “fracture,” and “scapular fracture.” The specific search strategy for PubMed is detailed in Table 1. After removing duplicates using EndNote and conducting an initial title/abstract screening, we applied strict inclusion and exclusion criteria to assess full-text articles, ultimately identifying 8 confirmed cases of scapular fracture caused by electrical injury (the screening process is illustrated in Fig. 3).

**Table 1:** Search strategy for PubMed database.

NO	Search items
#1	Electric Injuries [MeSH]
#2	electrical injury* [Title/Abstract]
#3	electric shock* [Title/Abstract]
#4	Electrocution [Title/Abstract]
#5	lightning injury* [Title/Abstract]
#6	lightning strike* [Title/Abstract]
#7	electrical trauma* [Title/Abstract]
#8	alternating current* [Title/Abstract]
#9	AC [Title/Abstract]
#10	DC [Title/Abstract]
#11	current [Title/Abstract]
#12	ampere [Title/Abstract]
#13	amperage [Title/Abstract]
#14	voltage [Title/Abstract]
#15	volt* [Title/Abstract]
#16	high tension [Title/Abstract]
#17	high voltage [Title/Abstract]
#18	low voltage [Title/Abstract]
#19	#1 OR #2 OR #3 OR #4 OR #5 OR #6 OR #7 OR #8 OR #9 OR #10 OR #11 OR #12 OR #13 OR #14 OR #15 OR #16 OR #17 OR #18
#20	Fractures, Bone [MeSH]
#21	Shoulder Fractures [MeSH]
#22	fractur* [Title/Abstract]
#23	dislocat* [Title/Abstract]
#24	subluxat* [Title/Abstract]
#25	broken bone [Title/Abstract]
#26	scapular fracture* [Title/Abstract]
#27	scapula fracture* [Title/Abstract]
#28	fracture of scapula [Title/Abstract]
#29	shoulder blade fracture* [Title/Abstract]
#30	#20 OR #21 OR #22 OR #23 OR #24 OR #25 OR #26 OR #27 OR #28 OR #29
#31	#19 AND #30

Through a review of the existing literature, we have substantiated and refined the core findings of this study from 2 distinct aspects. First, regarding both the incidence and the anatomical site, we observed that although fractures/dislocations resulting from low-voltage electrical shocks are overall uncommon,^[5]^ they demonstrate a distinct predilection for the upper limb, particularly the humerus and scapula (Fig. 3). The 2 cases of isolated scapular body fractures presented in this report align with this distribution pattern, supporting the assertion that the scapula is a key target for low-voltage electrical injury-related fractures. Second, concerning the injury characteristics, a pooled analysis of our 2 cases and 8 previously reported cases (Table 2) revealed that none of the scapular fractures caused by low-voltage electrical injury were associated with shoulder dislocation, and only 2 cases involved the glenoid cavity.^[6–13]^ This finding suggests that the primary clinical manifestation of such injuries may be the fracture itself, rather than joint dislocation or articular involvement.

**Table 2:** Case summary of scapular fractures due to low-voltage electrical injuries.

Author	AC/DC	Voltage (V)	Frequency (Hz)	Exposure time (s)	Injured side	Fracture location	Scapular dislocations	Involvement of joints	Associated fractures	Treatment
Tarquinio et al^[6]^	AC	440	60	Several seconds	Both	Body and glenoid base	No	Yes	No	Nonoperative
David R. et al^[7]^	AC	440	60	30–60	Both	Body and glenoid base	No	Yes	No	Nonoperative
Dumas J L et al^[8]^	AC	220	50	–	Both	Body and neck	No	No	No	Nonoperative
Dave D J et al^[9]^	–	–	–	–	Both	Glenoid	No	Yes	No	Nonoperative
Kotak B P et al^[10]^	AC	240	50	15–20	Both	Bilateral extra-articular fractures	No	No	No	Nonoperative
Rana M et al^[11]^	–	–	–	≤5–10	Right	Body	No	No	No	Nonoperative
Wen-Cheng et al^[12]^	AC	110	60	–	Left	Bilateral extra-articular fractures	No	No	No	Nonoperative
Michael B.C et al^[13]^	AC	230–240	50	–	Left	Body and glenoid	No	Yes	No	Operative
Present case	AC	220	50	4–5	Right	Body	No	No	No	Nonoperative
	AC	220	50	4–5	Left	Body	No	No	No	Nonoperative

In cases with undocumented voltage, the scenario (e.g., household appliances or residential wiring etc) clearly indicated low-voltage alternating current.

AC = alternating current; DC =direct current.

These characteristics stand in sharp contrast to scapular fractures resulting from traditional high-energy trauma (e.g., motor vehicle collisions and falls from a height), which are frequently associated with joint dislocations and other multiple injuries,^[14]^ with the fractures themselves predominantly located in the scapular body.^[15]^ In contrast, scapular fractures caused by low-voltage electrical injuries are confined to the scapula itself, typically sparing adjacent joints and causing no trans-articular damage. Given the remarkable concentration of injury energy within the scapular structure itself, presenting solely as fracture, we further investigated whether low-voltage electrical injuries exhibit a predilection for specific fracture locations. For this purpose, we examined all 10 cases (Table 2) and applied the Hardegger classification. The results demonstrated that such fractures also predominantly affect the scapular body, with only a minority involving the glenoid cavity. This distribution pattern aligns with the predilection site of high-energy trauma-induced fractures. It can thus be concluded that regardless of the injury mechanism (be it high-energy trauma or low-voltage electrical injury) and irrespective of the underlying cause, scapular fractures exhibit a consistent predilection for the scapular body.

**Figure 4.**
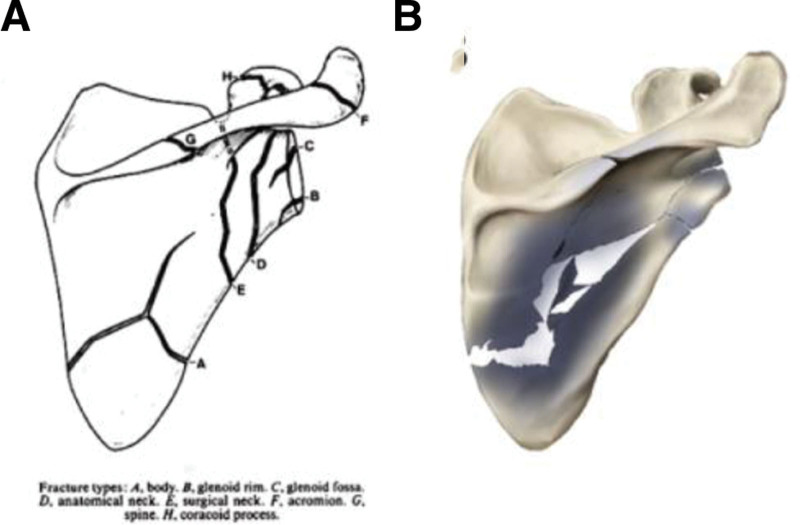
Fracture types of scapulae adapted from Hardegger et al^[19]^ (A). Simulation image of scapular fractures after electric injury. The deeper the color, the greater the fracture incidence (fractures occurred most frequently in the body, and relatively less frequently in the glenoid, scapular neck, and scapular spine) (B).

This commonality raises a pivotal question: in the absence of an external high-energy impact, why does the distribution of fractures resulting from low-voltage electrical injuries still resemble that of high-energy trauma? We hypothesize that the mechanism is related to the unique anatomical structure of the scapula, the typical entry path of the current via the upper limb, and the inherent properties of the electrical current itself. The scapula is a large and thin bone, with its body and inferior angle being particularly mechanically vulnerable. When the hand contacts a low-voltage alternating current, the induced tetanic muscle contraction forces the muscles attached to the inferior and lower part of the scapula to generate sustained,^[16,17]^ powerful outward and downward traction forces, while the muscles attached to the mid-superior part produce opposing inward and upward traction forces.^[18]^ These sustained, multidirectional antagonistic forces acting on the biomechanically weak region of the scapula can directly lead to fracture, with the fracture line potentially extending to the glenoid cavity and scapular neck (Fig. 4). It must be emphasized that the above mechanism is currently only a hypothesis, and its validation requires further rigorous experimental investigation and biomechanical analysis.

Rana et al^[11]^ pointed out that although AC can lead to coma, cardiac arrest, etc, low-frequency AC often causes sustained muscle contractions and arrhythmias. This also explains why there were no other organic injuries except scapular fracture and mild myoglobin increases in the 2 reported cases. By reviewing the cases of scapular fractures secondary to electric injuries, we found that these fractures were mostly caused by low-frequency AC (<60 Hz), with short exposure times and voltages mainly in the range of 110 to 440V. Moreover, traumas mostly occur in the body and lower-middle parts of the scapula and rarely injure the joints. Domestic power supplies generally exhibit the aforementioned features. Therefore, when approaching such cases, we should be alert to the occurrence of scapular fractures in patients with shoulder pain but without posterior dislocation. For most scapular fractures, conservative treatment such as analgesia, fixation, and progressive physical therapy can usually achieve satisfactory clinical results. Furthermore, early radiographic, CT, or magnetic resonance imaging examinations will be helpful for diagnosis.

In summary, even though scapular fracture is not a common complication of electric injury, upon receiving such cases, clinicians should not only give priority to the comprehensive assessment and treatment of life-threatening injuries but also be vigilant about the possibility of scapular fracture in patients with shoulder pain but no posterior dislocation.

Despite the small sample size of studies based on this direction and the limited data that can be gathered, there are still traces to follow. Even if our current efforts have not yielded ground-breaking theoretical results, we hope to attract our colleagues’ attention to such cases.

## Author contributions

**Conceptualization:** Jia-Qi Jia, Hong-Jun Liu, Kang Geng.

**Data curation:** Jia-Qi Jia, Hong-Jun Liu, Kang Geng.

**Formal analysis:** Jia-Qi Jia, Hong-Jun Liu, Jun-Chao Dai, Kang Geng.

**Funding acquisition:** Kang Geng.

**Investigation:** Jia-Qi Jia, Hong-Jun Liu, Kang Geng.

**Methodology:** Jia-Qi Jia, Hong-Jun Liu, Kang Geng.

**Project administration:** Jia-Qi Jia, Hong-Jun Liu, Kang Geng.

**Resources:** Jia-Qi Jia, Hong-Jun Liu, Jun-Chao Dai, Kang Geng.

**Software:** Jia-Qi Jia, Hong-Jun Liu, Kang Geng.

**Supervision:** Jia-Qi Jia, Hong-Jun Liu, Kang Geng.

**Validation:** Jia-Qi Jia, Hong-Jun Liu, Kang Geng.

**Visualization:** Jia-Qi Jia, Hong-Jun Liu, Jun-Chao Dai, Kang Geng.

**Writing – review & editing:** Jia-Qi Jia, Hong-Jun Liu, Kang Geng.

**Writing – original draft:** Jia-Qi Jia, Kang Geng.
